# Do fever-relieving medicines have anti-COVID activity: an *in silico* insight

**DOI:** 10.2217/fvl-2020-0398

**Published:** 2021-03-24

**Authors:** Fahad Hassan Shah, Kyeong Ho Lim, Song Ja Kim

**Affiliations:** 1 ^1^Department of Biological Sciences, College of Natural Sciences, Kongju National University, Gongju 32588, Republic of Korea; 2 ^2^Department of Construction and Environmental Engineering, College of Engineering, Kongju National University, Cheonan 31080, Republic of Korea

**Keywords:** acetaminophen, antiviral activity, drug repositioning, naproxen, nonsteroidal anti-inflammatory drugs, novel coronavirus-19, prediction of activity spectra for substances

## Abstract

**Aim:** The present study was performed to determine the inhibitory interaction of fever-relieving medicines with severe acute respiratory syndrome coronavirus-2 (SARS-CoV-2) essential proteins. **Materials & methods:** Structure-based drug repositioning was performed using PYRX 0.9 and these drugs were directed toward the predicted active site of SARS-CoV-2 spike glycoprotein receptor-binding domain, main protease and RNA-dependent RNA polymerase. **Results:** Results showed that acetaminophen and naproxen have considerable inhibitory activity and show a high affinity for active residues of these proteins. The prediction of activity spectra for substances (PASS) studies showed that these drugs are anti-inflammatory, antiviral and immunostimulant. **Conclusion**: Hence, it is proven that these drugs have antiviral activity against SARS-CoV-2 and can stimulate the immune and anti-inflammatory response against this disease.

The pandemic storm led by novel coronaviruses infected billions and has claimed millions of lives worldwide. Infectious waves across different countries have begun at a rapid pace with increased lethality while therapeutics and vaccines are still awaiting approval from higher authorities. Coronaviruses are a group of highly contagious viruses that have their roots in order Nidovirales, family Coronaviridae and genus Beta coronaviruses [1]. Severe acute respiratory syndrome coronavirus-2, also known as 2019 novel coronavirus (COVID-19) is the member of beta-coronaviruses, which are positive-sense RNA viruses enclosed within enveloped capsid having a genome of about 30 kilobase pairs [2].

Upon infection with the host cell, the RNA genome of this virus directs the production of 27 viral proteins in which 16 nonstructural proteins are translated from 5′ end. Whereas, the structural region encodes four integral proteins contiguous to one another near 3′ end and are responsible for virus-host attachment (spike glycoprotein), capsid enveloping (envelope protein), membrane formation (M-protein) and genome encapsulation (nucleocapsid). The rest of the seven proteins called accessory proteins are translated through the process of ribosomal frameshifting. These viruses primarily affect the respiratory tract, disguising the immune system in order to proliferate viral copies [3]. Upon recognition, the immune system gets engaged in an exaggerated immunological response, also known as a cytokine storm that impairs the normal body physiological homeostasis, leading to death [4].

These viruses primarily affect the respiratory system by employing spike glycoproteins to interact with ACE receptors on the host cells and injecting their genome to initiate RNA translation and replication. During the initial stages, an individual infected with this virus remains asymptomatic for a definite time period because of immunosuppression facilitated by nonstructural proteins [3]. Upon immunorecognition, individual experiences nonspecific clinical symptoms, in other words, body pain, chills, fever and coughing. As the disease progresses, headache, olfactory dysfunction, diarrhea and later the development of a cytokine storm, which damages pulmonary cells and leads to acute respiratory distress and multiorgan failure.

During the manifestation of these clinically important symptoms, usually, fever-relieving medicines are prescribed and administered to alleviate fever and myalgia. Most patients develop immunity against this disease with these medicines, whereas other patients require extra supportive care to eradicate this infection [5]. So, it was hypothesized that fever medicines not only tackle fever but also perform antiviral activity by improving the immune response and inhibiting SARS-CoV-2 replication [6]. To elucidate this unexplained biological activity of fever medicine, the current research study was designed to determine the anti-Covid activity of fever-relieving medicines using an *in silico* screening platform.

## Materials & methods

### Receptors & ligands preparation

Critical SARS-CoV-2 proteins responsible for replication (SARS-Cov-2 RNA-dependent RNA polymerase (PDB ID: 6M71), viral–host interaction (SARS-CoV-2 receptor-binding domain (PDB ID: 7JMO) and viral polyprotein processing (SARS-CoV-2 main protease (PDB ID: 6LU7) were selected and procured from the RCSB protein databank (Supplementary Table 1). These protein receptors were initially prepared by removing pre-existing ligands and water ions from them through the Discovery Studio program and then minimized with Modrefiner [7]. Fever-alleviating drugs listed in (Table 1) were obtained from PubChem and refined with the PRODRG Server [8].

**Table 1.** List of fever-relieving medicines used against SARS-CoV-2 protein targets.

N	DRUGS	Pubchem CID
1	Acetaminophen	1983
2	Acetylsalicylic acid	2244
3	Ibuprofen	3672
4	Mefenamic acid	4044
5	Nimesulide	4495
6	Naproxen	156391
7	Diclofenac Sodium	5018304
8	Piroxicam	54676228

### Active site prediction

In order to predict the probable inhibitory interaction of ligands with corresponding receptors, active site residues play an integral role in substrate processing and inhibition of protein function. The interaction of ligands with these active residues halt substrate processing, which results in protein inhibition. By keeping this strategy in mind, DogSite Scorer [9] and Prank Web [10] were used to predict the active site residues within these receptors to direct these ligands toward these areas in the screening analysis.

### Drug repositioning procedure

Both minimized and refined versions of receptors and ligands were loaded on PYRX 0.9 software to facilitate the interaction of these drugs with the predicted active site residues of SARS-CoV-2 receptors (Supplementary Table 2). The algorithm selected for docking interaction between receptor-ligands was Autodock 4.2, which provides highly accurate results resembling *in vitro* results.

### PASS analysis & compound pharmacokinetics

Screened ligands SMILES (Supplementary Table 3) was used to predict important biological activities using the prediction of activity spectra for substances database (PASS) [11]. The pharmacokinetic prediction was performed to evaluate the efficacy and absorption, distribution, metabolism, excretion and toxicity (ADMET) qualities of these ligands through ADMET SAR 2.0 [12] and pKCSM [13].

## Results

### Drug repurposing results

Structure-based drug repositioning was performed that uses docking algorithm to target ligands toward a directed area for interaction on the receptor. The selected ligands, (Table 1) were focused on the active interface of SARS-CoV-2 receptors. These receptors were spike glycoprotein receptor binding domain (RBD), main proteases (M^PRO^) and RNA-dependent RNA polymerase (RdRp) enzyme implicated in the processing of viral polyproteins, virus–host interaction and viral replication. Among seven widely exploited fever-relieving drugs, acetaminophen and naproxen had favorable hydrogen bonding with the targeted active site of these receptors with binding energy of ~4.5–6.2 kcal/mol, as shown in (Table 2).

**Table 2.** Docking interaction of ligands with SARS-CoV-2 receptors.

N	Receptors	Ligands	Active site subunits predicted by DoGSiteScorer and Prank Web	Chemical bonding between receptor and ligands	Functional groups participating in hydrogen bond formation	Binding energy (Kcal/mol)	Ligand efficiency (Kcal/mol)	Inhibition constant (Ki)	LigRMSD calculation (Å)
1	SARS-CoV-2 main protease (6LU7)	Acetaminophen (Pubchem CID: 1983)	HIS41, MET49, PHE140, GLY143, CYS145, HIS164, GLU166, LEU167, PRO168, GLN189, THR190, ALA191	MET165, GLU166, VAL186	Hydroxyl and amide groups	-4.5	0.40	487.06 μM	1.87
		Naproxen (Pubchem CID: 156391)		GLY143, SER144, CYS145	Carboxylic group	-6.1	0.35	256.76 μM	1.44
2	SARS-CoV-2 receptor binding domain (7JMO)	Acetaminophen (Pubchem CID: 1983)	LEU273, GLY274, GLN275, GLY276, CYS277, PHE 278, GLY279, VAL281, ALA293, LYS295, LEU297, MET302, PHE307, MET314, VAL323, LEU325, ILE336, THR338, TYR340, MET341, SER342, GLY344, SER345, ASP348, ASP386, ARG388, ALA390, ASN391, LEU393, ALA403, ASP404, GLY405, LEU407, ALA408, ILE411, TYR416, LYS423, PRO425	PHE338, GLY339, LEU368	Hydroxy and amide groups	-5.7	0.51	109.51 μM	2.64
		Naproxen (Pubchem CID: 156391)		CYS336. PHE338, ASP364	Carboxylic group	-6.2	0.36	104.87 μM	2.21
3	SARS-Cov-2 RNA-dependent RNA polymerase (6M71)	Acetaminophen (Pubchem CID: 1983)	LYS47, TYR129, HIS133, ASP135, ASN138, ASN705, SER709, THR710, GLY774, LYS780, ASN781, SER784	PHE45, HIS133, ASN705	Hydroxy and Amide group	-6.1	0.55	134.97 μM	3.11
		Naproxen (Pubchem CID: 156391)		GLN468, ASN705	Carboxylic Group	-6.2	0.37	76.89 μM	2.89

#### SARS-CoV-2 main protease (M^PRO^)

**Figure 1.**
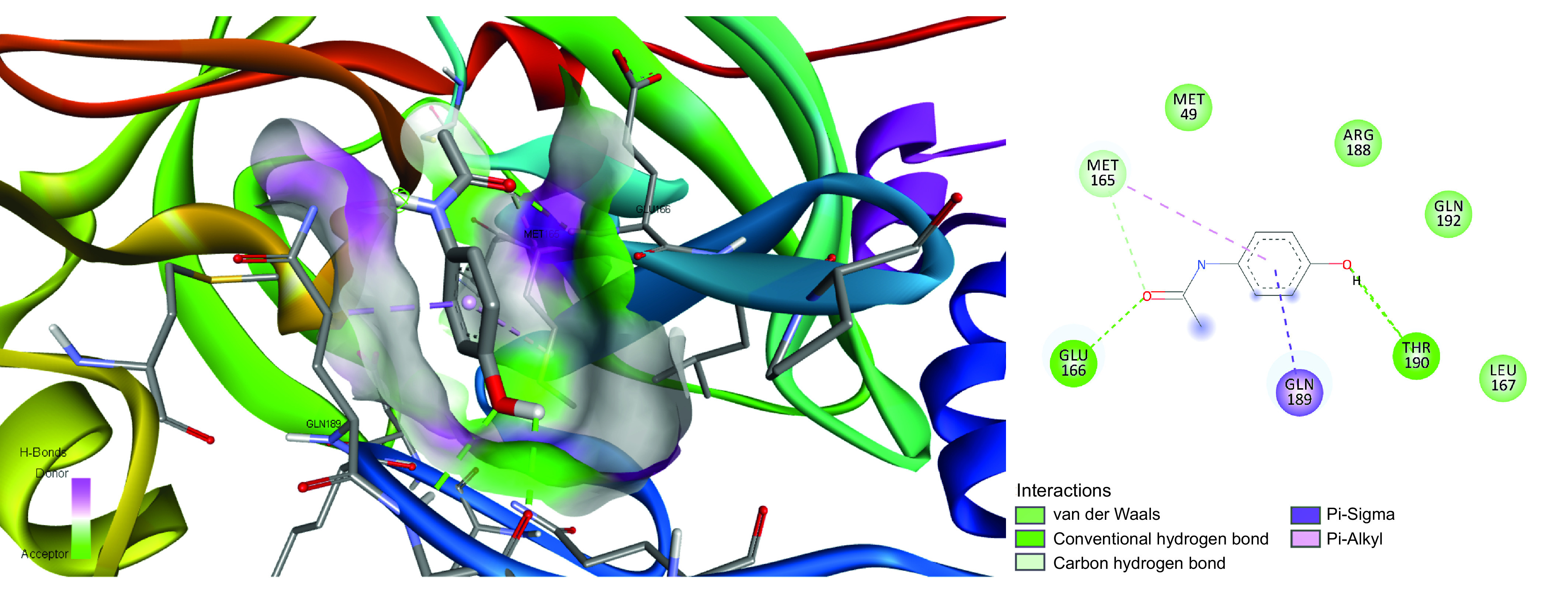
Binding pattern and functional groups exploited by acetaminophen to interact with SARS-CoV-2 main protease.

Hydroxyl and amide group of acetaminophen formed hydrogen bonds with MET165, GLU166 and VAL186 (Figure 1), whereas MET165 and GLN189 participated in hydrophobic interactions with the benzene group. The carboxylic group of naproxen established interactions with GLY143, SER144 and CYS145, and hydrophobic interaction made by the benzene group with CYS145, and MET165 (Supplementary Figure 1).

#### SARS-CoV-2 spike glycoprotein (RBD)

Acetaminophen established three hydrogen bonds with PHE338, GLY339 and LEU368 whereas benzene ring participated in forming hydrophobic interactions with PHE338 and VAL367 (Supplementary Figure 2). Naproxen employed carboxylic group to form hydrogen bonds with CYS336, PHE338 and ASP364 and benzene rings and oxy-group established hydrophobic interactions with VAL367, PHE338, LEU368 and PHE374 (Supplementary Figure 3).

#### SARS-CoV-2 RNA polymerase enzyme (RdRp)

Three hydrogen interactions were formed by three separate functional groups (hydroxyl, amino and carboxyl) of acetaminophen with PHE45, HIS133 and ASN705. Naproxen exploited the carboxylic group to form two hydrogen bonds with GLN468 and ASN705 and benzene rings and oxy-group formed hydrophobic interactions with PHE45, LEU708, TYR129 and ALA34, respectively (Supplementary Figures 4 & 5).

### Ligands toxicity behavior

The toxic behavior of acetaminophen and naproxen was determined in rodent models by administering various doses through four administration routes. It was observed that acetaminophen incites adverse and organ-damaging effects, when the quantity of the drug is increased to 2,000,000 mg/kg orally, 100,000 mg/kg intravenously and 700,000 mg/kg and 1,100,000 mg/kg for the intraperitoneal and subcutaneous route. For naproxen, acute toxicity recorded for intraperitoneal route was 500,000 mg/kg; 200,000 mg/kg for intravenous route; 900,000 mg/kg oral; and 600,000 mg/kg for subcutaneous. OECD chemical classification results placed acetaminophen in Class 5 chemicals and naproxen in Class 4, respectively. The organ-damaging effects (Supplementary Table 4) and adverse effects of these drugs are summarized in (Table 3).

**Table 3.** Lethal dosage of ligands and its corresponding adverse and organ damaging effects.

N	Compounds	LD50 value for intraperitoneal route (mg/kg)	LD50 value for intravenous route (mg/kg)	LD50 value for oral route (mg/kg)	LD50 value for subcutaneous route (mg/kg)	Chemical classification by OECD project	Adverse effects	Organ-specific damage
1	Acetaminophen (Pubchem CID: 1983)	714,200	100,400	2,629,000	1,132,000	Class 5	Nephrotoxicity, myocardial infarction, cardiac failure	Urinary bladder, kidney, stomach, hematopoietic system, thyroid gland and ovaries
2	Naproxen Naproxen (Pubchem CID: 156391)	502,900	233,500	907,100	606,000	Class 4	Hepatotoxicity, myocardial infarction, cardiac failure, nephrotoxicity	Skin, urinary bladder and kidney

### PASS & pharmacokinetic prediction of ligands

The activity of acetaminophen and naproxen was analyzed with thousands of biologically active substrates to discover new effects and mechanisms, which was facilitated by PASS online server. The biological activities predicted by PASS algorithm are 90 % synonymous with *in vitro* experiments [11]. Results showed that acetaminophen is an antiviral agent, active against influenza, picornavirus, adenovirus, coronavirus, HIV and herpes virus. This provides invaluable justification that acetaminophen is involved in subduing viral proliferation activity. Also, they are immunostimulant and inhibit the activity of transcription factor NF-κB. Naproxen is active against rhinoviruses, picornaviruses and influenza and dampens the response of NF-κB and IL-6, which are implicated in the cytokine storm (Table 4).

**Table 4.** Biological activity of ligands predicted by PASS online.

N	Compounds	Pa	Pi	Biological activity
1	Acetaminophen (Pubchem CID: 1983)	0.588	0.014	Antiviral (Influenza)
		0.551	0.032	Antiviral (Picornavirus)
		0.473	0.009	Antiviral (Adenovirus)
		0.400	0.004	^†^HIV-2 reverse transcriptase inhibitor
		0.342	0.009	^†^3C-like protease (Human coronavirus) inhibitor
		0.342	0.013	Transcription factor NF kappa B inhibitor
		0.370	0.059	Immunostimulant
		0.310	0.048	RNA synthesis inhibitor
		0.307	0.056	Antiviral (Poxvirus)
		0.310	0.076	^†^HCV IRES inhibitor
		0.312	0.083	Antiviral (Herpes)
2	Naproxen (Pubchem CID: 156391)	0.870	0.005	Anti-inflammatory
		0.860	0.003	Antipyretic
		0.642	0.006	Nonsteroidal anti-inflammatory agent
		0.449	0.049	Antiviral (Rhinovirus)
		0.361	0.146	Antiviral (Picornavirus)
		0.342	0.010	Interleukin 6 antagonist
		0.335	0.015	^†^Transcription factor NF kappa B inhibitor
		0.327	0.110	Analgesic
		0.313	0.081	Antiviral (Influenza)

† 3C-like protease: 3-Chymotrypsin-like cysteine protease; HCV IRES: Hepatitis C virus Internal Ribosome Entry Site; HIV: Human immunodeficiency virus; NF kappa B: Nuclear Factor Kappa B.

Both of these drugs have high intestinal absorption equipped with increased blood–brain barrier (BBB) penetration capability and oral bioavailability, except for acetaminophen, which lacks oral bioavailability. Subcellular localization of these drugs are mitochondria and are nonreactive toward P-glycoprotein. Acetaminophen possesses affinity for CYP2C9 substrates and naproxen for CYP2C9 and inhibits CYPE1A2. These drugs are noncarcinogenic and nonmutagenic. Naproxen has low water solubility and high plasma protein binding, whereas acetaminophen has high water solubility and low plasma protein-binding affinity (Table 5).

**Table 5.** Pharmacokinetic analysis of screened ligands.

Properties	Compounds	
	Acetaminophen (Pubchem CID: 1983)	Naproxen (Pubchem CID: 156391)
**Absorption**		
Human intestinal absorption	High +	High +
^†^Caco-2	+	+
Water solubility (log mol/l)	-1.131 (soluble)	-4.098 (highly soluble)
Plasma protein binding	0.372	1.052
**Distribution**		
P-glycoprotein substrate	-	-
P-glycoprotein I inhibitor	-	-
P-glycoprotein II inhibitor	-	-
^†^BBB permeability	+	+
Oral bioavailability	-	+
Sub-cellular localization	Mitochondria	Mitochondria
**Metabolism**		
^†^CYP3A4 substrate	-	-
CYP2C9 substrate	+	+
CYP2D6 substrate	-	-
CYP3A4 inhibition	-	-
CYP2C9 inhibition	-	-
CYP2C19 inhibition	-	-
CYP2D6 inhibition	-	-
CYP1A2 inhibition	-	+
**Excretion**		
Total clearance (log ml/min/kg)	0.493	0.193
Renal OCT2 substrate	No	No
**Toxicity**		
^†^AMES toxicity	No	No
Hepatotoxicity	No	No
^†^*hERG* inhibition^†^	No	No
Eye irritation	No	No
Carcinogenicity	No	No

† AMES: *Salmonella typhimurium* reverse mutation assay; BBB: Blood–brain barrier; Caco-2: Colorectal adenocarcinoma cells; CYP: Cytochromes P450; hERG: human ether-a-go-go related gene; OCT: Organic cation transporter 2.

## Discussion

Asymptomatic disease nature, uncontrollable inflammatory response and delayed immunological reactions are typical manifestations of novel coronavirus infection. These manifestations are caused by nonstructural proteins processed by SARS-CoV-2 main proteases [3], whereas replication and virus–cell interactions are facilitated by RdRp and spike protein [1,14]. Currently, these targets are being exploited for vaccine and drug design and development studies. Several different drug candidates have been introduced from various screening, *in vitro* and clinical studies in the past few months. Drugs approved by the US FDA for the treatment of novel coronavirus in critical patients are remdesivir and barictinib [15]. However, their dosages must be calibrated for an effective response against this virus and prevent deleterious side effects. The mild symptomatic cases are dealt with fever relieving medicines to alleviate fever and other symptoms to buy enough time for the host body to develop an immune response to ward off this infection. Interestingly, 80% of patients recover from this disease by taking these fever relieving medicines [5]. Being analgesic, anti-inflammatory and antipyretic agent, these drugs are somehow acting as an antiviral agent in this disease, which remained elusive until now. To unravel this mystery, we have performed a drug repositioning study to check the inhibitory interaction of these fever-relieving medicines against SARS-CoV-2 essential proteins. It was observed that only two commonly exploited drugs (acetaminophen and naproxen) successfully established hydrogen interaction with the active site residues of these receptors with adequate binding energy, and ligand efficiency. Furthermore, root-mean-square deviation (RMSD) and inhibitory constant values favored these drugs. PASS prediction studies revealed that acetaminophen has moderate antiviral activity against different viruses including coronaviruses and low immunostimulant activity. On the other hand, naproxen has strong anti-inflammatory activity and avert the proliferation of rhinoviruses, picornaviruses and influenza viruses and inhibit NF-κB and IL-6 activity.

These activities suggest that, in the early phase of SARS-CoV-2 infection, these drugs will perform antiviral activity by reducing viral entry by binding to the RBD of spike glycoprotein, modulate adaptive immune response and ameliorate viral clearance by dampening the viral polyprotein processing and replication. These drugs will also prevent SARS-CoV-2-induced damage to pulmonary epithelial cells and other organs implicated in the pulmonary phase. Our findings are in consonance with Terrier *et al.*, 2020 [16] preprint, who infected Vero E6 cell and 3D reconstituted human airway epithelia with SARS-CoV-2 to evaluate the antiviral and pulmonary protective activity of naproxen. In their study, naproxen dose-dependently reduced viral titers in both cell lines and induced a protective effect against viral-induced damage. Naproxen also reduces IL-6 and NF-kB levels as evident from the study by Kang *et al.*, 2001 [17], which can be useful in reducing cytokine storm and organ damage during hyper-inflammation phase. However, these results are specific for naproxen, whereas the role of acetaminophen remains elusive in this disease, but can be explored by employing various *in vitro* experiments to confirm these *in silico* predicted activities.

Toxicity and pharmacokinetic studies were performed to evaluate the toxicity and pharmacokinetic properties of these drugs, to equip scientists with valuable information about these drugs that will help them devise various approaches to amplify its antiviral and anti-inflammatory response in animal and clinical trials. Acetaminophen has high acute toxicity values with mild adverse effects but possesses low oral bioavailability. Naproxen has comparatively low acute toxicity than acetaminophen, with life-threatening side effects. Both toxic and bioavailability issues can be resolved with controlled drug-release properties [18], drug-delivery nanocarriers [19] and drug-dose calibration [20].

## Conclusion

To the best of our knowledge, our study predicted the antiviral mechanism of acetaminophen and naproxen for the first time. These drugs are highly effective against essential SARS-CoV-2 protein targets and possess moderate toxicity and pharmacokinetic properties. However, more studies are essential to validate the findings of this research in animals and clinical trials. The findings of this study have opened a new and safer gateway for using these drugs as a prophylactic and antiviral medication that can be of great significance to clinicians and biomedical scientists struggling to curb the ongoing pandemic.

**Summary points**

- SARS-CoV-2, also known as novel coronavirus-19 (COVID-19) is a highly contagious and life-threatening disease and employs spike glycoprotein, main proteases and RNA-dependent RNA polymerase enzyme to interact with angiotensin-converting enzyme-2 receptors on host cells, process viral polyproteins and replicate to produce viral copies of themselves inside the infected cell.
- Most patients infected with this virus recover and develop immunity against this disease by taking fever relieving medicines and supportive care.
- Structure-based drug repositioning studies confirmed that acetaminophen and naproxen act as antiviral, anti-inflammatory and immunostimulatory agents against this disease.
- These drugs interact with active ligand binding site residues of spike glycoprotein, main protease and RdRp enzyme.
- Further biological activities predicted by PASS indicate that these drugs are active against coronavirus and various other pathogenic viruses and can prevent the onset of cytokine storm.
- Toxicity and pharmacokinetic properties of these drugs are cautionary but can be ameliorated with drug delivery, controlled drug release and drug dose calibration methods.
- To the best of our knowledge, this study is the first of its kind to describe the antiviral activity of acetaminophen and naproxen against essential SARS-CoV-2 receptors.
