# Supplementary material for: Do fever-relieving medicines have anti-COVID activity: an in silico insight
Source: Future Virol. 2021 Mar 24:10.2217/fvl-2020-0398. doi: 10.2217/fvl-2020-0398 (PMC7989381; doi:10.2217/fvl-2020-0398)
Supplement: Supplementary file 1 [file supplementary_file.docx]

**
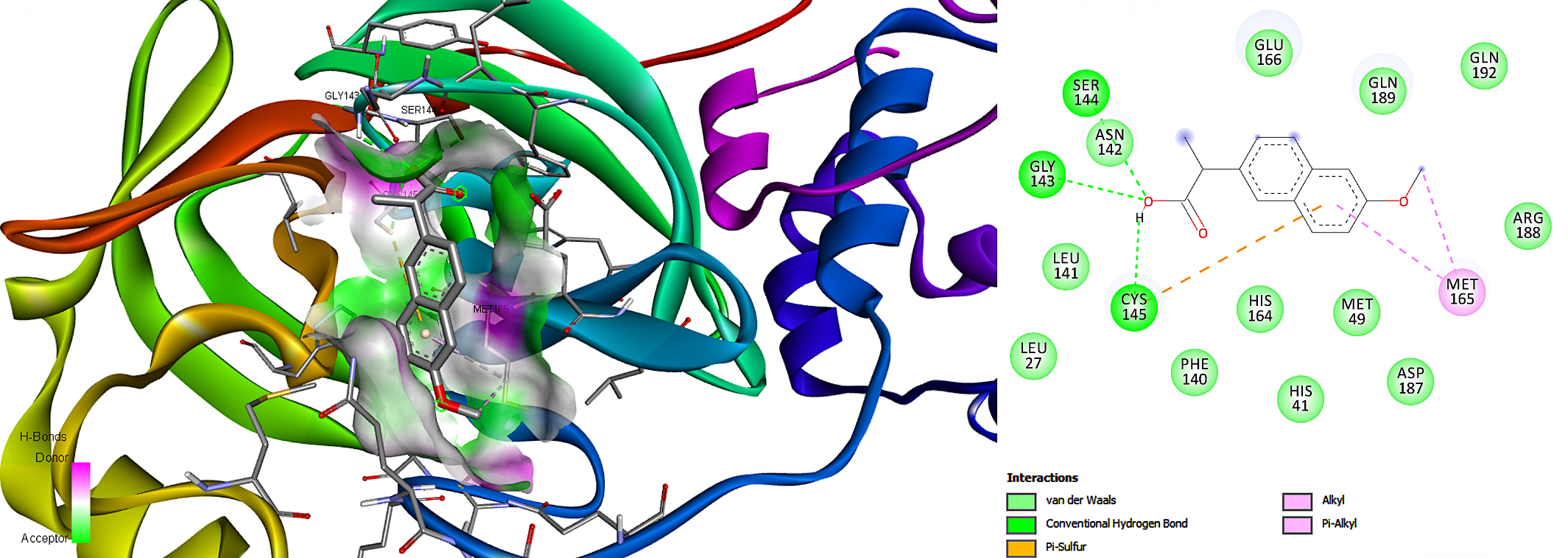
**

**Supplementary Figure 1.** Binding Pattern and Functional groups exploited by Naproxen to interact with SARS-CoV-2 Main protease

**
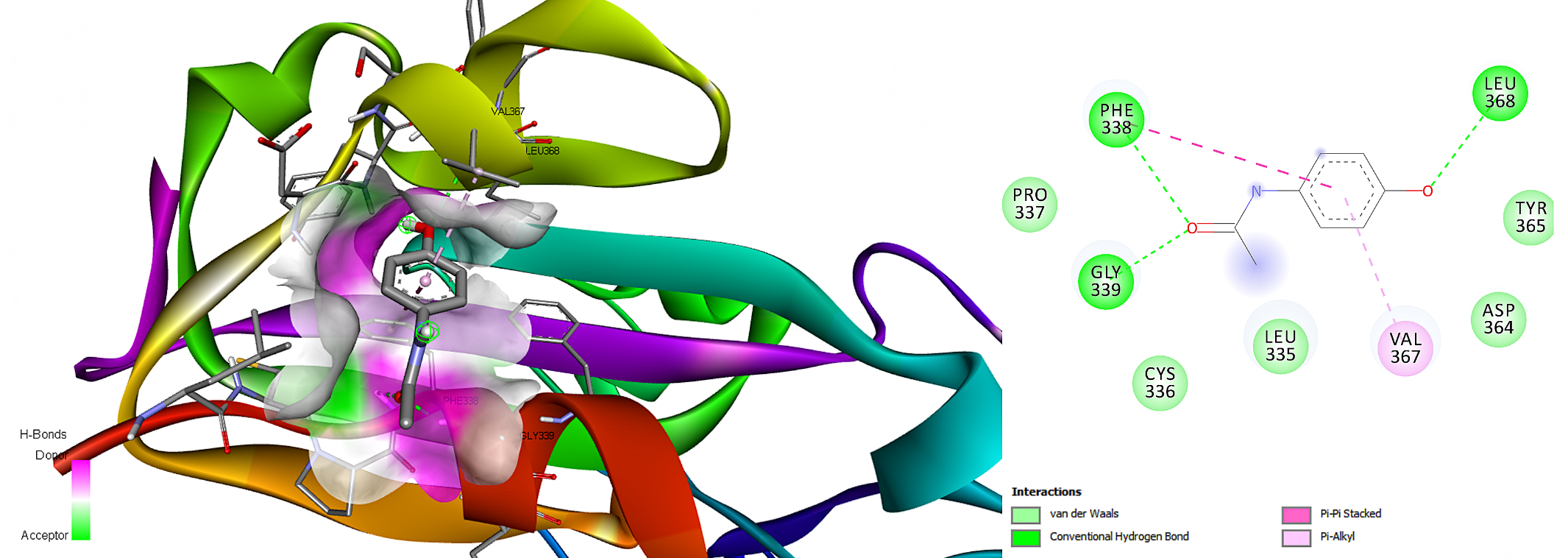
**

**Supplementary Figure 2.** Binding Pattern and Functional groups exploited by Acetaminophen to interact with SARS-CoV-2 Spike Glycoprotein Receptor Binding Domain

**
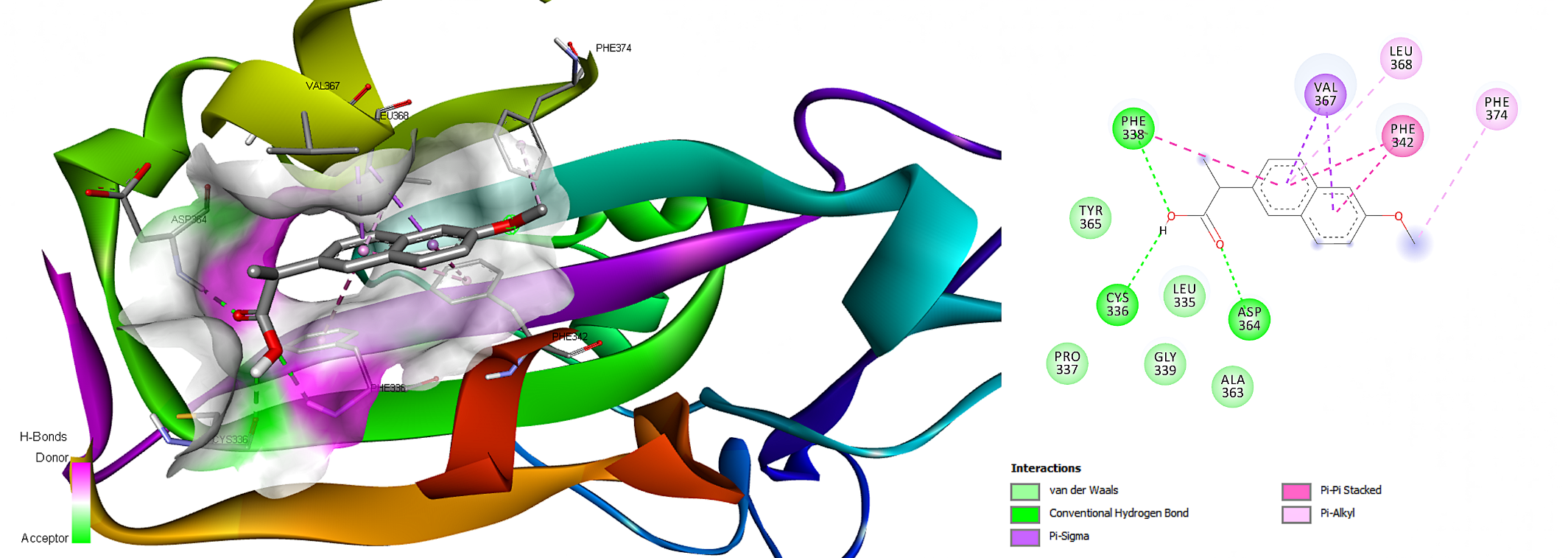
**

**Supplementary Figure 3.** Binding Pattern and Functional groups exploited by Naproxen to interact with SARS-CoV-2 Spike Glycoprotein Receptor Binding Domain.

**
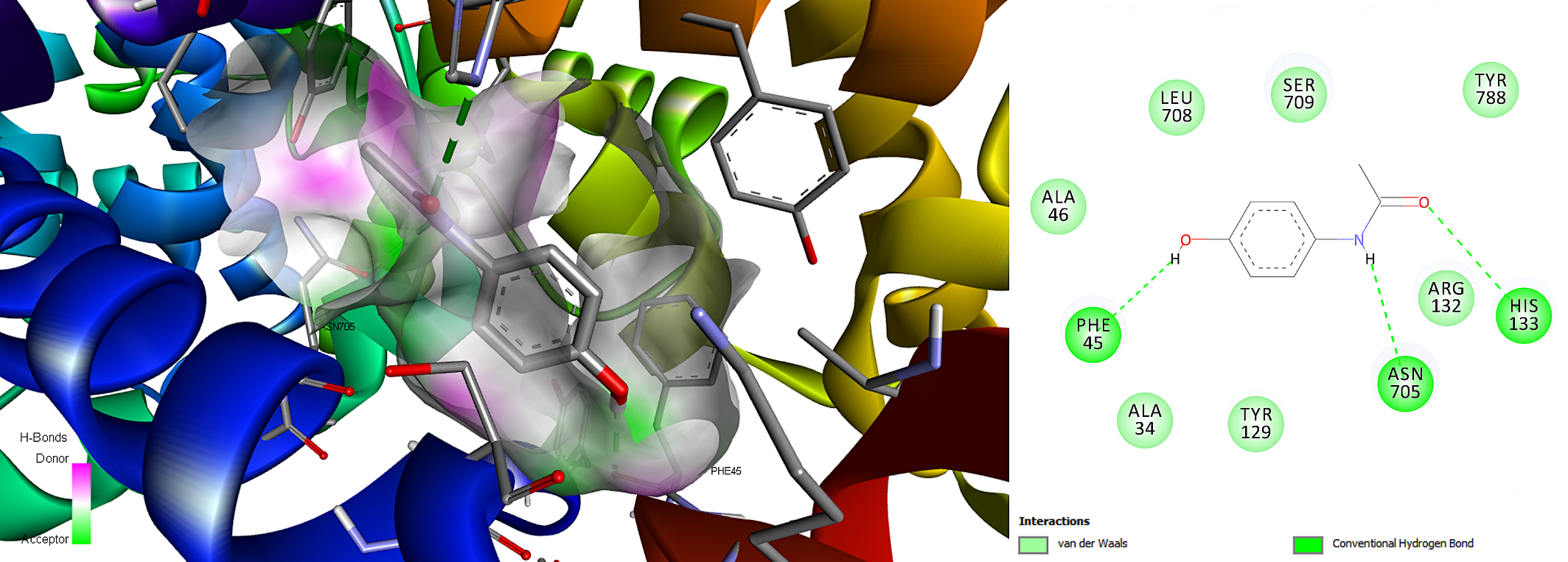
**

**Supplementary Figure 4.** Binding Pattern and Functional groups exploited by Acetaminophen to interact with SARS-CoV-2 RNA dependent RNA Polymerase Enzyme

**Supplementary Figure 5.** Binding Pattern and Functional groups exploited by Naproxen to interact with SARS-CoV-2 RNA dependent RNA Polymerase Enzyme
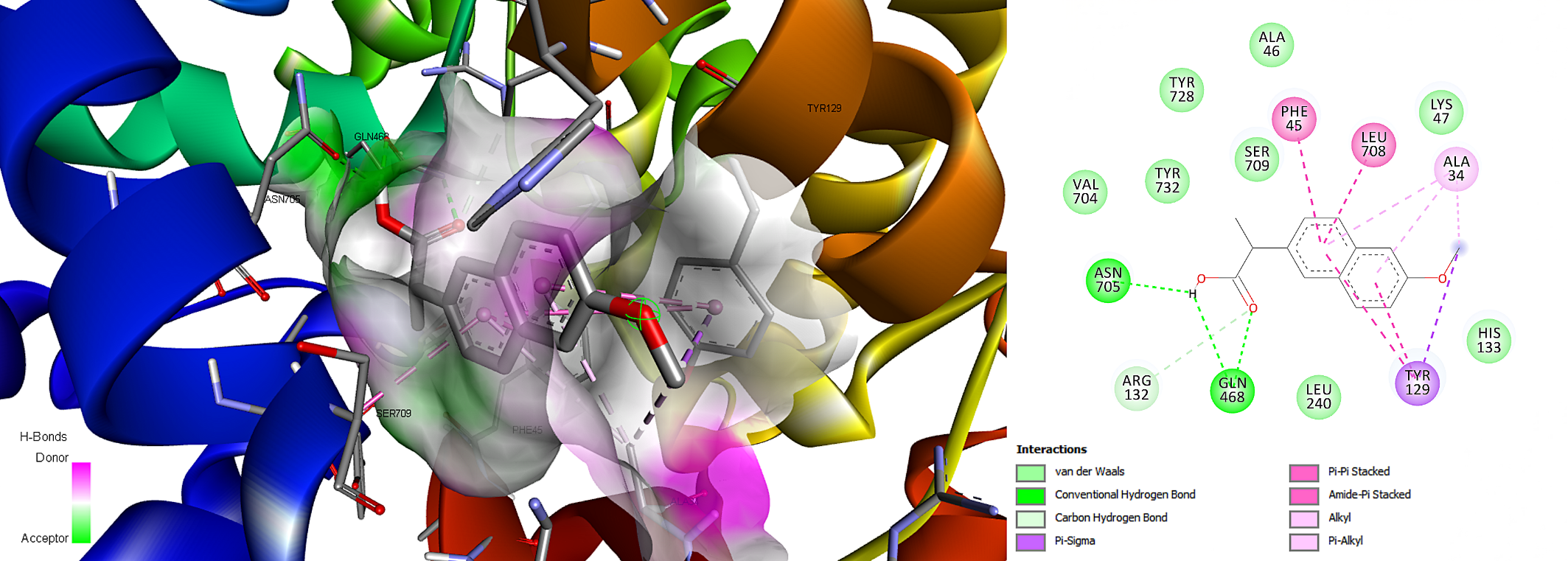


**Supplementary Table 1.** Protein Sequences and Chains Selected for Docking Interaction

| **No** | **Receptor** | **RCSB Protein databank Code** | **Protein Chain** | **Protein Sequence** |
| --- | --- | --- | --- | --- |
| 1 | COVID-19 Main Protease | 6LU7 | Chain A | SGFRKMAFPSGKVEGCMVQVTCGTTTLNGLWLDDVVYCPRHVICTSEDMLNPNYEDLLIRKSNHNFLVQAGNVQLRVIGHSMQNCVLKLKVDTANPKTPKYKFVRIQPGQTFSVLACYNGSPSGVYQCAMRPNFTIKGSFLNGSCGSVGFNIDYDCVSFCYMHHMELPTGVHAGTDLEGNFYGPFVDRQTAQAAGTDTTITVNVLAWLYAAVINGDRWFLNRFTTTLNDFNLVAMKYNYEPLTQDHVDILGPLSAQTGIAVLDMCASLKELLQNGMNGRTILGSALLEDEFTPFDVVRQCSGVTFQ |
| 2 | SARS-CoV-2 receptor binding domain | 7JMO | Chain A | RVQPTESIVRFPNITNLCPFGEVFNATRFASVYAWNRKRISNCVADYSVLYNSASFSTFKCYGVSPTKLNDLCFTNVYADSFVIRGDEVRQIAPGQTGKIADYNYKLPDDFTGCVIAWNSNNLDSKVGGNYNYLYRLFRKSNLKPFERDISTEIYQAGSTPCNGVEGFNCYFPLQSYGFQPTNGVGYQPYRVVVLSFELLHAPATVCGPKKSTNLVKNKCVNFSGHHHHHH |
| 3 | SARS-Cov-2 RNA-dependent RNA polymerase | 6M71 | Chain A | SADAQSFLNRVCGVSAARLTPCGTGTSTDVVYRAFDIYNDKVAGFAKFLKTNCCRFQEKDEDDNLIDSYFVVKRHTFSNYQHEETIYNLLKDCPAVAKHDFFKFRIDGDMVPHISRQRLTKYTMADLVYALRHFDEGNCDTLKEILVTYNCCDDDYFNKKDWYDFVENPDILRVYANLGERVRQALLKTVQFCDAMRNAGIVGVLTLDNQDLNGNWYDFGDFIQTTPGSGVPVVDSYYSLLMPILTLTRALTAESHVDTDLTKPYIKWDLLKYDFTEERLKLFDRYFKYWDQTYHPNCVNCLDDRCILHCANFNVLFSTVFPPTSFGPLVRKIFVDGVPFVVSTGYHFRELGVVHNQDVNLHSSRLSFKELLVYAADPAMHAASGNLLLDKRTTCFSVAALTNNVAFQTVKPGNFNKDFYDFAVSKGFFKEGSSVELKHFFFAQDGNAAISDYDYYRYNLPTMCDIRQLLFVVEVVDKYFDCYDGGCINANQVIVNNLDKSAGFPFNKWGKARLYYDSMSYEDQDALFAYTKRNVIPTITQMNLKYAISAKNRARTVAGVSICSTMTNRQFHQKLLKSIAATRGATVVIGTSKFYGGWHNMLKTVYSDVENPHLMGWDYPKCDRAMPNMLRIMASLVLARKHTTCCSLSHRFYRLANECAQVLSEMVMCGGSLYVKPGGTSSGDATTAYANSVFNICQAVTANVNALLSTDGNKIADKYVRNLQHRLYECLYRNRDVDTDFVNEFYAYLRKHFSMMILSDDAVVCFNSTYASQGLVASIKNFKSVLYYQNNVFMSEAKCWTETDLTKGPHEFCSQHTMLVKQGDDYVYLPYPDPSRILGAGCFVDDIVKTDGTLMIERFVSLAIDAYPLTKHPNQEYADVFHLYLQYIRKLHDELTGHMLDMYSVMLTNDNTSRYWEPEFYEAMYTPHTVLQHHHHHHHH |

**Supplementary Table 2** Autodock 4.2 Docking Settings for Receptor-Ligand Interaction

| **No** | **Receptor** | **Center** | | | **Dimension Å** | | |
| --- | --- | --- | --- | --- | --- | --- | --- |
|  |  | **X** | **Y** | **Z** | **X** | **Y** | **Z** |
| 1 | COVID-19 Main Protease | -18.6902 | 23.4523 | 61.8673 | 28.4351 | 27.42.9385 | 30.5708 |
| 2 | SARS-CoV-2 receptor binding domain | -84.4897 | 15.8490 | 6.7545 | 34.0839 | 32.8534 | 25.000 |
| 3 | SARS-Cov-2 RNA-dependent RNA polymerase | 134.1625 | 127.0021 | 94.5429 | 38.0465 | 52.5467 | 41.3416 |

**Supplementary Table 3** Fever Relieving Medicine Canonical SMILES

| **No** | **Compounds** | **PUBCHEM CID** | **Canonical SMILES** |
| --- | --- | --- | --- |
| 1 | Acetaminophen | 1983 | CC(=O)NC1=CC=C(C=C1)O |
| 2 | Naproxen | 156391 | CC(C1=CC2=C(C=C1)C=C(C=C2)OC)C(=O)O |

| **No** | **Compounds** | **Pa** | **Pi** | **Organ Affected (Male Rat)** | **Organ Affected (Female Rat)** |
| --- | --- | --- | --- | --- | --- |
| **1** | Acetaminophen | 0.920 | 0.015 | urinary bladder | **-** |
|  |  | 0.861 | 0.051 | kidney | Urinary Bladder |
|  |  | 0.639 | 0.125 | **-** | hematopoietic system |
|  |  | 0.635 | 0.060 | **-** | ear Zymbals gland |
|  |  | 0.506 | 0.133 | stomach | **-** |
|  |  | 0.457 | 0.137 | nasal cavity | **-** |
|  |  | 0.424 | 0.200 | - | Liver |
|  |  | 0.323 | 0.320 | - | thyroid gland |
|  |  | 0.333 | 0.165 | skin | **-** |
| **2** | **Naproxen** | 0.399 | 0.297 | urinary bladder | urinary bladder |
|  |  | 0.361 | 0.288 | stomach | **-** |
|  |  | 0.300 | 0.213 | skin | **-** |

**Supplementary Table 4**. Effect of Fever Relieving Drugs on Rat’s Various Organs
